# Effect of Tartary Buckwheat Bran Substitution on the Quality, Bioactive Compounds Content, and *In Vitro* Starch Digestibility of Tartary Buckwheat Dried Noodles

**DOI:** 10.3390/foods11223696

**Published:** 2022-11-18

**Authors:** Chaoqiang Xue, Xiaona Guo, Kexue Zhu

**Affiliations:** 1 State Key Laboratory of Food Science and Technology, Jiangnan University, 1800 Lihu Avenue, Wuxi 214122, China; 2 School of Food Science and Technology, Jiangnan University, 1800 Lihu Avenue, Wuxi 214122, China

**Keywords:** Tartary buckwheat bran, noodles, quality, digestibility, starch structure, enzyme activity

## Abstract

This study aimed to investigate the impact of partial replacement of Tartary buckwheat flour (TBF) with Tartary buckwheat bran flour (TBBF) on the quality, bioactive compounds content, and *in vitro* starch digestibility of Tartary buckwheat dried noodles (TBDNs). When the substitution of TBBF was increased from 0 to 35%, the cooking and textural properties decreased significantly (*p* < 0.05), while the content of bioactive compounds (phenolic, flavonoids and dietary fiber) increased significantly (*p* < 0.05). In addition, the substitution of TBBF decreased the starch digestibility of TBDNs. A 10.4% reduction in eGI values was observed in the TBDNs with 35% TBBF substitution compared to the control sample. The results of differential scanning calorimetry showed that with the increase of TBBF, TBDNs starch became more resistant to thermal processing. Meanwhile, the X-ray diffraction and Fourier transform infrared spectroscopy results revealed that the long- and short-range ordered structures of TBDN starch increased significantly (*p* < 0.05). Furthermore, the substitution of TBBF decreased the fluorescence intensity of α-amylase and amyloglucosidase. This study suggests that replacing TBF with TBBF could produce low glycemic index and nutrient-rich TBDNs.

## 1. Introduction

Noodles, one of the traditional staple foods in China, are becoming popular all over the world, among which, dried noodles are becoming more and more popular in the staple food market, due to their advantages, such as the convenience of consumption, easy storage, and relatively low price [1]. In recent years, the incidence of obesity and diabetes has been increasing globally. Nearly 537 million people suffer from diabetes, and this number will reach 700 million by 2045 [2]. As a wheat-based food, noodles are rich in starch, which can cause a rapid increase in blood glucose. Obviously, wheat noodles can no longer meet the criteria of a low glycemic index (GI) diet.

Tartary buckwheat is rich in bioactive compounds, such as phenolic, flavonoids, and dietary fiber [3]. As is well known, phenolic and flavonoids are helpful in reducing the risk of chronic diseases such as diabetes, cardiovascular disease, and certain cancers [4]. Dietary fiber benefits human health, and a diet high in dietary fiber can prevent type 2 diabetes [5]. In addition, Tartary buckwheat protein is high in lysine, which is the most restricted amino acid in wheat [6]. Consequently, Tartary buckwheat is anexcellent functional ingredient to improve human health and has been used to produce foods such as cookies, buckwheat-rice noodles, and pasta [7,8,9]. Recently, Tartary buckwheat flour has become popular as a partial substitute for wheat flour in the production of noodles, due to its nutritional properties [10]. 

Tartary buckwheat bran is the outermost layer of buckwheat seeds, containing more bioactive compounds compared to Tartary buckwheat flour (TBF) [11]. Rachman et al. [12] reported the total phenolic and flavonoids content increase caused by the substitution of buckwheat bran, which reduced the digestibility of buckwheat gel. Thus, enriching wheat-based noodles with Tartary buckwheat bran allows the development of low GI noodles. However, it is usually discarded in the production of refined flour or used as animal feed, due to its coarse taste and poor processing performance [13].

There are adverse effects of Tartary buckwheat bran on the quality of noodles, including reduced texture properties and sensory acceptability [14]. Gluten dilution, physical hindrance, and a poor gluten network caused by competitive water absorption by buckwheat bran are the main factors responsible for the deterioration of noodle quality [15]. Current research has mainly focused on the influence of different treatments on the physicochemical properties of Tartary buckwheat bran [13,16]. In contrast, little attention had been paid to the effect of Tartary buckwheat bran on the starch digestibility of noodles.

Thus, the aim of the present study was to investigate the influence of TBBF on the quality, bioactive compound content, and *in vitro* starch digestibility of TBDNs. The study especially focused on the mechanism by which TBBF affected the digestion of TBDNs. It was hypothesized that TBBF would influence the starch digestibility of TBDNs in two ways: (i) the bioactive compounds (including phenolic, flavonoids and dietary fiber) in the TBBF might interact with starch, affecting the structure of starch molecules; (ii) the inhibition of digestive enzymes by TBBF. To the best of our knowledge, this is the first time the effect of TBBF on the digestibility of TBDNs through the evolution of starch structure and inhibition of digestive enzymes has been studied. To verify our hypotheses, the thermal properties, crystallinity (long-range), and short-range ordered structure of TBDN starch were investigated. Meanwhile, the fluorescence quenching of α-amylase and amyloglucosidase was also studied.

## 2. Materials and Methods

### 2.1. Materials and Chemicals

Tartary buckwheat flour (TBF) (the content of moisture, protein, fat, and ash was 14.22%, 12.31%, 2.42%, and 1.63%, respectively) and Tartary buckwheat bran flour (TBBF) (the content of moisture, protein, fat, and ash was 13.26%, 19.40%, 3.58% and 2.80%, respectively) were purchased from Liangshan Jianmao Food Co., Ltd. (Xichang, China). Wheat flour (the content of moisture, protein, fat, and ash was 12.60%, 15.01%, 0.63%, and 0.43%, respectively) was obtained from COFCO Flour (Haining) Co., Ltd. (Jiaxing, China). Wheat gluten (the content of moisture, protein, fat, and ash was 7.11%, 72.79%, 0.85%, and 0.84%, respectively) was obtained from Binzhou Zhongyu Food Co., Ltd. (Binzhou, China). The α-amylase (10080, from hog pancreas 50 U/mg), amyloglucosidase (10115, from *Aspergillus niger* 110 U/mg), pepsin (P7000, from porcine gastric mucosa, ≥250 U/mg), and pancreatin (P7545, from porcine pancreas, 8 × USP) were purchased from Sigma-Aldrich Chemical Co. (St. Louis, MO, USA). A Total Dietary Fiber kit was purchased from Megazyme International Ireland Ltd. (Bray, Ireland). All chemical reagents used were of analytical grade.

### 2.2. Preparation of Tartary Buckwheat Dried Noodles (TBDNs)

As seen in Table 1, TBDNs were manufactured using Tartary buckwheat flour (TBF) and wheat flour, in a proportion of 70:30 (*w*/*w*). In addition, TBBF was used to substitute TBF in the ratios of 0%, 5%, 15%, 25% and 35%, respectively. To form a dough sheet, the blended (100 g) flour and wheat gluten (7 g) were mixed evenly in a mixer (JMHZ-200, Beijing, China) for 6 min. Then, distilled water (37.5 mL) was added and mixed for 5 min. To distribute the moisture evenly, the dough was sealed and rested in a plastic bag (25 °C, 75% RH). After that, the dough was sheeted using a laboratory semi-automatic noodle machine (JMTD-168/140, Beijing, China). The roller gap was gradually decreased from 2.2 mm to 0.8 mm with a reduction of 0.2 mm (dough sheets were pressed three times at each roller gap), and then the dough sheets were cut into strips with a width of 2.0 mm. Finally, the noodles were dried in a laboratory intelligent drying platform (SYT-030, Beijing, China). The drying procedure was gradually changed from low temperature and high humidity (30 °C, 80% RH) to relatively high temperature and low humidity (45 °C, 60% RH), until the moisture content of noodles reached 12%.

### 2.3. Quality Analysis of TBDNs

#### 2.3.1. Cooking Quality Analysis of TBDNs

Cooking properties, including water absorption and cooking loss, were characterized using the AACC method 66-50 [17]. Briefly, 10 g TBDNs were cooked in 500 mL deionized water till the optimal cooking time, which was defined as the time when the bright yellow core inside the Tartary buckwheat dried noodles was observed have disappeared, by cutting a cross-section of the noodles. Tap water was used for rinsing the cooked noodles. After that, the surface water was absorbed with filter paper and left for 30 s. The cooking water was collected and diluted to 500 mL with deionized water. Then, 100 mL of the cooking water was transferred to a weighted and dried beaker. Finally, the beaker was put on the induction cooker, to evaporate most of the water, and dried in an oven (130 °C) till at constant weight. The water absorption was defined as the weight ratio of the TBDNs before and after cooking. The cooking loss was defined as the weight ratio of the residue in the beaker to the raw TBDNs (dry weight).

#### 2.3.2. Texture Quality Analysis of TBDNs

The textural properties of TBDNs were characterized using a TA-XT2i Texture Analyzer (Stable Micro Systems, Surrey, UK) based on the method described by Zou et al. [10]. Three cooked TBDNs were fixed to the platform and compressed with a P/36R cylindrical probe at 75% strain and 5.0 g trigger force. The pre-test, test, and post-test speed were all 1.0 mm/s. In addition, the tensile characteristics of the noodles were measured using an A/SPR probe. A noodle was wrapped around the probe, then stretched at a starting distance of 100 mm and a trigger force of 5.0 g. The pre-test, test, and post-test speeds were 1.0 mm/s, 1.0 mm/s, and 10 mm/s, respectively.

### 2.4. Estimation of Total Phenolic and Flavonoid Content

#### 2.4.1. Extraction of Phenolic and Flavonoids from TBDNs

According to Biney et al. [8], freeze-dried raw and cooked TBDN samples (0.5 g) were accurately weighed into a tube, and 20 mL of acidified methanol (HCl/methanol/water, 1:80:20, *v*/*v*/*v*) was used to extract the phenolic and flavonoids. The mixture was shaken for 3 h in a water bath (30 °C, 200 rpm). Then, the supernatant was removed, and another 20 mL of acidified methanol was added for repeat extraction. After that, the extracts were combined and centrifuged at 4000× *g* for 15 min. The supernatant was collected and placed in a round bottom flask for evaporation. Finally, the supernatant was diluted to 10 mL with the same acidified methanol for further use.

#### 2.4.2. Total Phenolic Content

The total phenolic content (TPC) of TBDNs was measured according to the study of Dulf et al. [18]. First, Folin–Ciocalteu reagent was diluted 10-fold and 2.5 mL was transferred to a centrifuge tube. Then, the supernatant (0.5 mL) was added and incubated for 6 min. After that, Na_2_CO_3_ solution (2 mL, 7.5%) was added to neutralize the reaction. Finally, the reaction solution was placed in an incubator (25 °C, 1 h). The absorbance of samples was determined at 760 nm using a Microplate Reader (EPOCH2, BioTek, Winooski, VT, USA). The standard curve was made using the gallic acid, and the TPC was denoted by the gallic acid equivalents (mg GAE/g DW).

#### 2.4.3. Total Flavonoids Content

According to the method of aluminum chloride colorimetric assay [19], the supernatant (1 mL) was transferred to a tube, NaNO_2_ (3 mL, 5%) and AlCl_3_ (3 mL, 10%) were added to the solution, respectively, and incubated (25 °C, 6 min). Then NaOH (2 mL, 1 M) solution was used to create the alkaline environment required for the reaction. Then the reaction solution was diluted to 10 mL with deionized water, shaken well, and incubated at 25 °C for 30 min. The absorbance of samples was determined at 510 nm using a Microplate Reader (EPOCH2, BioTek, Winooski, VT, USA). The standard curve was constructed using rutin, and the TFC was denoted by rutin equivalents (mg RE/g DW).

### 2.5. Determination of Dietary Fiber

Dietary fiber content, including total (TDF), insoluble (IDF), and soluble dietary fiber (SDF) of TBDNs were measured by the enzyme gravimetric method using a Megazyme Dietary Fiber kit, according to the AOAC method 991.43 [20].

### 2.6. Analysis of In Vitro Starch Digestion

According to Fan et al. [21], the *in vitro* simulation digestion procedure was divided into oral, gastric, and intestinal. HCl, NaOH, and various salt solutions were used to create the digestive environments corresponding to the three phases. First, TBDNs were cooked for the optimal cooking time and cut into a width of 2 mm, and accurately weighed to 5 g into a centrifuge tube (50 mL). Then, 4 mL of simulated salivary fluid (SSF) was added and vortexed thoroughly. After that, the NaOH (20 μL, 1 M) solution was used to adjust the pH to 7. Deionized water (955 μL) with dissolved α-amylase and CaCl_2_ (25 μL, 0.3 M) solution were used to bring the volume of the suspensions to 10 mL. The activity of α-amylase in the suspensions from the oral phase was 750 U. Second, after shaking for 5 min (200 r/min), deionized water (1.995 mL), CaCl_2_ (5 μL, 0.3 M) solution, and 8 mL of the simulated gastric fluids (SGF) with dissolved pepsin were added. The activity of pepsin in the suspensions from the gastric phase was 40,000 U. Third, after shaking (200 r/min, 2 h), the NaOH (230 μL, 1 M) solution was used to adjust the pH to 7 for the pancreatic enzyme reaction. Bile salt (2.5 mL), CaCl_2_ (40 μL, 0.3 M) solution, and 16 mL of simulated intestinal fluid (SIF) were mixed with the suspensions from the gastric phase. Then, the mixture was diluted to 40 mL with deionized water. The pancreatic enzyme content was determined by the activity of amylase in the suspension (40 mL), with an activity of 8000 U. Finally, the suspension was continuously shaken (200 r/min), and 0.1 mL aliquots were transferred for measurement after 0, 10, 20, 30, 60, 90, 120, 180, 240, and 300 min. To stop the reaction, 0.4 mL of anhydrous ethanol was added. Before centrifugation (10,000× *g*, 15 min), the mixture was rested for 40 min. A glucose assay kit (Nanjing Jiancheng Bioengineering Institute, Nanjing, China) was used to measure the glucose content, which was converted into the amount of digested starch. Furthermore, the starch digestion rate was calculated as the total starch content divided by the amount of digested starch. 

The starch hydrolysis curves of TBDNs were made based on a nonlinear first-order rate equation:

(1) $$C_{t}{=C}_{\infty }\left(1\;-{\;e}^{-\mathrm{kt}}\right),$$

Parameter C_t_ represents the starch hydrolysis rate at time t; parameter C_∞_ represents the predicted maximum hydrolysis rate; and parameter k represents the starch hydrolysis constant. As described by Zou et al. [22], noodles contain two starch fractions, which have different hydrolysis rates. The LOS plot (Equation (2)) was transformed from Equation (1), elaborating the linear relationship between ln(dC_t_/dt), and t was used to analyze the hydrolysis rates of different fractions of starch. Equation (2) was given, as follows:

(2) $$\ln\left({\mathrm{dC}}_{t}/\mathrm{dt}\right)=-\mathrm{kt}+\ln\left(C_{\infty }k\right),$$

The parameter k is used to indicate the rate of starch hydrolysis. The AUC (area under hydrolysis curve), HI (hydrolysis index), eGI (estimated glycemic index), and RDS (rapidly digested starch is the amount of starch digested after 20 min), SDS (slowly digested starch is the amount of starch digested between 20 min and 120 min), RS (resistant starch is the amount of starch remaining after 120 min) were calculated using the method of Sun et al. [23].

### 2.7. Differential Scanning Calorimetry Measurements (DSC)

The thermal properties of TBDNs were analyzed using a DSC (DSC-3; Mettler Toledo, Zurich, Switzerland), according to the method of Han et al. [24]. First, samples and deionized water were weighed in an aluminum pan at a ratio of 1:3. Then, the pan was sealed under high pressure and equilibrated at 4 °C for 12 h. After this, samples and an empty pan (as a reference) were put into an oven. Then, the heating procedure was started, which increased from 30 °C to 100 °C at a ratio of 5 °C/min.

### 2.8. X-ray Diffraction Analysis (XRD)

The XRD patterns of starch in TBDNs were determined using an X-Ray diffractometer (D2 PHASER, Bruker AXS Inc., Karlsruhe, Germany) with Cu-Kα radiation (λ = 0.15406 nm), according to Han et al. [25]. The experimental parameters were as follows: operating voltage, 30 kV; current, 10 mA; scan range, 4° to 40° (2θ); scan rate, 3.6°/min; step size, 0.03°. The relative crystallinity and type of crystal were obtained using MDI Jade 6.0 software. 

### 2.9. FTIR Analysis 

The spectra of starch in TBDNs were determined using a FTIR spectrometer (Nicolet iS10, Thermo Nicolet, Madison, WI, USA). First, freeze-dried TBDNs flour was mixed with KBr in a proportion of 1:150. Then the mixture was pressed into transparent sheets, and loaded onto the ATR cell. After that, the spectra of 4000–400 cm^−1^ were scanned 32 times with a resolution of 4 cm^−1^. An empty cell was recorded as a background. In addition, the IR absorption intensity ratios at 1050 to 1022 cm^−1^ (R-1047/1022) and 1022 to 992 cm^−1^ (R-1022/992) were obtained using the method described by Liu et al. [1]. Briefly, the spectra in the range of 1200–800 cm^−1^ were baseline corrected, smoothed, and deconvoluted using omnic software (version 8.0) with the half-width and enhancement factors of 19 cm^−1^ and 2.1. 

### 2.10. Fluorescence Quenching Analysis 

The quenching effect of TBDNs on α-amylase and amyloglucosidase was measured using a fluorescence spectrometer (F-7000, Hitachi, Tokyo, Japan) according to Liu et al. [26]. Freeze-dried TBDNs flour (20 mg) was dissolved in sodium acetate buffer (0.2 M, 10 mL) with a pH of 6, then α-amylase (0.2 mg/mL, 2 mL) or amyloglucosidase (0.2 mg/mL, 2 mL) solution dissolved by the simulated intestinal fluid (SIF in Section 2.6) were added and incubated in a shaking incubator (37 °C, 220 rpm) for 30 min. Then, the mixture was centrifuged at 6000× *g* for 15 min. After that, the supernatant was transferred for the assay. Samples (1 mL) were scanned in the range of 300–400 nm, with a scanning rate of 1200 nm/min. The excitation wavelength, excitation slit width, and emission slit width were 288 nm, 5 nm, and 5 nm, respectively.

### 2.11. Statistical Analysis

All data are presented as means ± standard deviations. The statistical significance was analyzed using one-way ANOVA followed by Duncan’s test (*p* < 0.05) using SPSS 26. LOS and non-linear fitting of starch hydrolysis rate were performed using origin 2021.

## 3. Results and Discussion

### 3.1. Quality and Bioactive Compounds Content of TBDNs

#### 3.1.1. Cooking and Textural Properties

The cooking parameters of TBDNs are presented in Table 2. Water absorption represents the ability of noodles to absorb water. Cooking loss represents the amount of particles that diffuse from noodles into the water during cooking. Deficiency in water absorption could produce hard and coarse textured noodles, and a high cooking loss signifies a low cooking tolerance and high stickiness of noodles, which is undesirable in noodle production [27]. As the proportion of TBBF increased, the water absorption decreased from 132% to 119%, while the cooking loss increased from 4.64% to 6.53%, which indicated a deterioration of the cooking quality of the TBDNs [28]. This might have been due to the lower grinding level of TBBF, resulting in a lower broken starch content and more insoluble dietary fiber, which limited the water absorption of TBDNs during the cooking process [29]. In addition, dietary fiber hindered the development of the gluten network, preventing the starch from being completely encapsulated. As a result, the starch easily leached out during cooking, and thus led to a high cooking loss. This was consistent with the research of Liu et al. [14], who stated that the addition of common buckwheat bran hindered the development of the gluten network, resulting in low water absorption and high cooking loss. 

The textural properties of TBDNs were influenced by the substitution ratio of TBBF. As the substitution of TBBF was increased, the hardness and resilience values of TBDNs increased initially, but then decreased. According to Fan et al. [30], noodle hardness could be increased through the covalent and non-covalent interactions between protein and xylans, which resulted in high molecular mass polymers. However, when the amount of arabinoxylans exceeded a threshold, this interaction was reduced. When the content of TBBF exceeded 25%, the decrease in hardness and resilience was probably due to a serious collapse of the gluten network. Jin et al. [31] reported that bran fortification had a dilution effect on the gluten network, reducing the hardness of dried noodles. In addition, the competition of buckwheat bran for water absorption inhibited the cross-linking of gluten [15]. The values of cohesiveness, tensile strength, and elasticity decreased continuously, which indicated the deterioration of the TBDN tenacity. Han et al. [32] studied the effects of different components of Tartary buckwheat on the quality of noodles. They found that the higher the fiber content, the lower the tensile strength. Cao et al. [33] found that the tensile properties of noodles were positively correlated with the gluten quality. Thus, the weakening of the gluten network caused by the bran was responsible for the decrease in tensile strength and elasticity.

#### 3.1.2. Total Contents of Phenolic and Flavonoids

As seen in Table 3, the TPC and TFC of raw noodles increased significantly (*p* < 0.05), from 5.86 and 5.36 for the control to 12.32 and 7.95 for the noodles with 35% TBBF substitution (TBBF-35), respectively. Compared with raw noodles, both the TPC and TFC of cooked noodles decreased. Surprisingly, total phenolic and flavonoids retentions were highest in the noodles with 10% TBBF substitution (TBBF-10) and 15% TBBF substitution (TBBF-15), respectively. This was consistent with the results of the TBDN hardness. This was possibly because the dietary fiber filled in the gluten network, which helped encapsulate the bioactive compounds and protected them from thermal degradation. According to Fu et al. [7], the retention rate was related to the integration and continuous networkof starch. In addition, the retention of total phenolic was lower than that of total flavonoids. According to Sinkovič, L. et al. [34], TBBF contained more phenolic and flavonoids than TBF, and they were thermally unstable. Some phenolic and flavonoids of the noodles were degraded and lost in the noodle soup, due to the intense hydrothermal action [34]. The lower retention rate of phenolic was because it is more sensitive to heat than flavonoids [7].

#### 3.1.3. Dietary Fiber Content

In this study, the content of TDF, IDF, and SDF were determined and are presented in Table 3. It was reported that dietary fiber blocks the fat absorption and reduces energy intake, by regulating food intake, digestion, and absorption [35]. Thus, dietary fiber intake is associated with a reduced risk of the development of obesity and diabetes. As seen in Table 3, the TDF content in TBDNs was much higher than that of SDF. With the increase of TBBF, the TDF contents ranged from 3.47 to 9.92 g per 100 g, the IDF contents ranged from 2.55 to 8.48 g per 100 g, and the SDF contents ranged from 0.7 to 1.37 g per 100 g. The increase of dietary fiber in TBDNs was related to the higher dietary fiber content of Tartary buckwheat bran compared to Tartary buckwheat flour [36]. In addition, the dietary fiber of Tartary buckwheat bran was dominated by insoluble dietary fiber [37]. Combined with the results for quality, although the addition of TBBF weakened the tenacity of the TBDNs, there was little change in the hardness and resilience, and the bioactive compound (phenolic, flavonoids and dietary fiber) content of the TBDNs was increased significantly (*p* < 0.05).

### 3.2. In Vitro Starch Digestion

**Figure 1:**
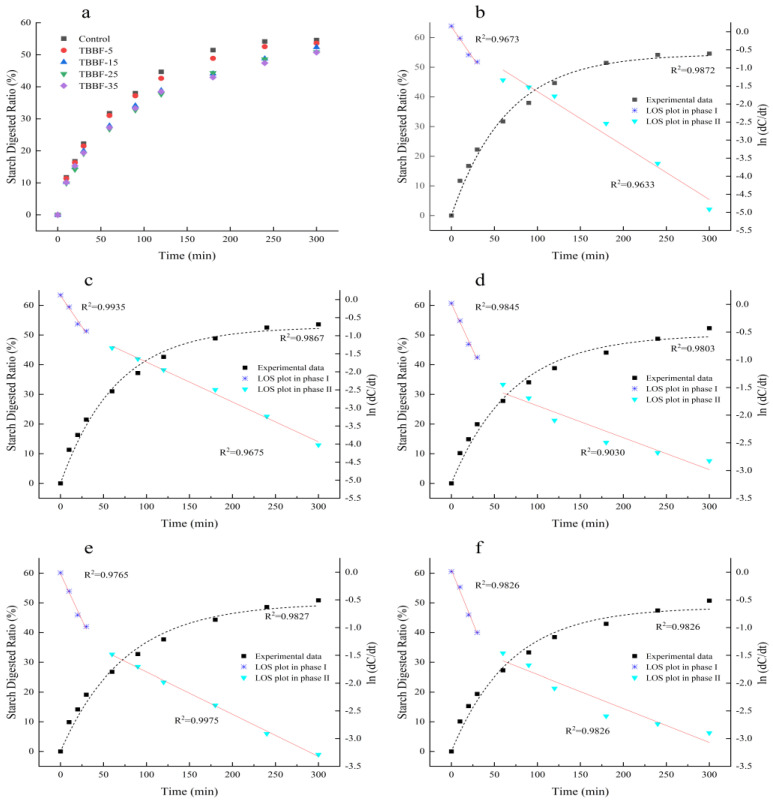
Digestograms for Tartary buckwheat dried noodles with different substitutions of Tartary buckwheat bran flour (**a**); (**b**–**f**) represent the LOS fitting digestion curves, which were substituted by Tartary buckwheat bran flour at the level of 0, 5%, 15%, 25%, and 35%. The LOS plots in phase Ι are characterized by blue, and phase ΙΙ is characterized by bright green. The black squares and dotted line represent the overall digestion data and model-fit curves. R^2^ represents the coincidence between the experimental data and the model-fit curves.

As shown in Figure 1, starch was digested rapidly in the first 30 min and slowly reached the maximum digestion after 180 min. In addition, the *in vitro* digestion curves of starch decreased with increasing levels of TBBF substitution (Figure 1a). For instance, the proportion of digested starch significantly (*p* < 0.05) decreased from 54.59% of the control to 50.73% with TBBF-35 when the digestion proceeded to 300 min. Table 4 shows the digestive parameters fit by the first-order nonlinear formula. With the addition of TBBF, the first-order digestion rate constant (k) and the calculated equilibrium starch hydrolysis (C_∞_) decreased significantly (*p* < 0.05), but k increased slightly when the substitution with TBBF reached 35%. Moreover, with increased addition of TBBF, the RDS and SDS dropped from 16.78% and 27.93% to 15.29% and 23.20%, respectively, while the RS increased significantly (*p* < 0.05), from 55.29% to 61.51%. These results suggest that the TBDNs starch was difficult to digest, due to the substitution of TBBF. This might have been because TBBF contains more dietary fiber and polyphenols, which reduced the digestibility of the TBDNs [16]. Noodles with an eGI below 55 are considered a low GI food [38]. Thus, all of the samples (TBBF-15, TBBF-25, and TBBF-35) meet this standard (Table 4). The logarithm of slope (LOS) plot is a linear relationship between time (t) and the logarithm of the digestion data (ln (dC_t_/dt)), and that reveals how the digestion rate changed during the digestion process [22]. To further investigate the influence of TBBF on the starch digestibility of TBDNs, all of the *in vitro* digestion curves of the starch were fit with the LOS. As seen in Figure 1b–f, the digestive curves for all TBDNs clearly showed two discontinuous parts. According to Zou et al. [22], the starch in the outer layer of the noodle was entirely gelatinized and could be hydrolyzed at a high rate. However, the rate was relatively low in the center area of noodles because it was tightly wrapped by the gluten matrix. Thus, k_1_ and k_2_ are the digestion rate constants for the two different starch fractions. Table 4 shows that k_1_ decreased slightly and then increased, while k_2_ significantly (*p* < 0.05) decreased from 1.50 to 0.61. The decrease of k_1_ and k_2_ indicates that the starch of TBDNs were more difficult to digest. In addition, the increase of k_1_ with the 35% TBBF substitution could have been due to the severe disruption of the gluten network, resulting in an increased exposure of starch to digestive enzymes [39]. This could also be demonstrated by the slight increase of RDS with TBBF-35. Fan et al. [21] demonstrated that the digestibility of starch was related to the gluten network. It was clear that the gluten network of the TBDNs was disrupted with the addition of TBBF. Therefore, the digestibility of the starch was expected to increase. However, the results showed a decrease in starch digestibility. Thus, the nature of the TBDNs starch might have been altered, leading the starch to become more resistant to enzymatic digestion. Moreover, with the addition of TBBF, the contents of total phenolic, flavonoids, and dietary fiber significantly (*p* < 0.05) increased (Table 3). According to Zhu et al. [40], starch formed complexes with phenolic compounds, in the form of single amylose helices promoted by hydrophobic interactions and hydrogen bonding, which decreased the digestibility of starch. Additionally, dietary fiber could reduce the activity of digestive enzymes, leading to a low digestibility of starch [41]. Overall, changes in starch structure and the inhibition of the enzymatic activity of digestive enzymes caused by the TBBF addition might have been responsible for the decrease in the digestibility of the TBDN starch.

### 3.3. Thermal Properties of TBDNs

Thermal characteristics of the TBDNs are shown in Table 5. With the replacement of TBBF, the onset temperature (T_o_) of TBDNs significantly increased, from 63.37 °C to 67.70 °C (*p* < 0.05), while the peak (T_p_) and conclusion temperature (T_c_) changed slightly. In addition, the enthalpy change of gelatinization (ΔH) gradually decreased from 3.66 J/g to 1.81 J/g. The increase of T_o_ was due to the dietary fiber hindering starch from contacting the water, which limited the swelling and gelatinization of starch [42]. In addition, the higher the crystallinity, the greater the resistance to gelatinization [43]. As shown in Table 3, the TBDNs with more TBBF contained more phenolic and flavonoids, which might have interacted with starch through hydrogen bonding, reducing the stability of the double helix of starch. It was found that tea polyphenols and sorghum extracts interacted with starch, which reduced the enthalpy of starch [44,45]. In addition, the substitution of TBBF decreased the total starch content of TBDNs, resulting in a lower enthalpy change (ΔH). Similarly, Molina et al. [46] reported that the enthalpy change (ΔH) was reduced in cookies, because the enhancement of bran negatively influenced the gelatinization.

### 3.4. XRD Results of TBDNs

**Figure 2:**
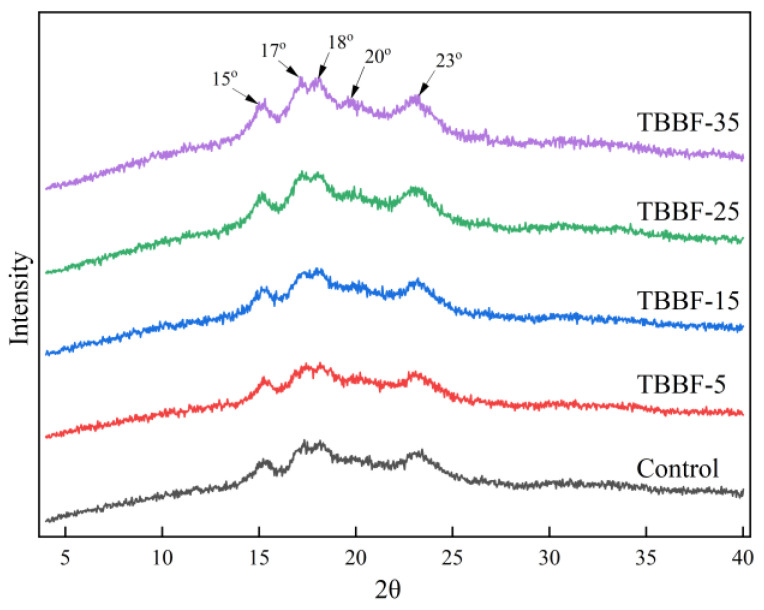
XRD patterns of Tartary buckwheat dried noodles with different substitutions of Tartary buckwheat bran flour. Control, TBBF-5, TBBF-15, TBBF-25, and TBBF-35 represent Tartary buckwheat dried noodles with 0%, 5%, 15%, 25%, and 35% substitution of Tartary buckwheat bran flour, respectively.

The XRD results of all TBDNs are presented in Figure 2. All TBDNs showed A-type diffraction patterns, characterized by peaks at 15°and 23° (2θ), and with adjacent double peaks at 17° and 18° (2θ). In addition, there was a small peak at 20° (2θ), which was the crystallization peak of the complex formed by amylose, with other fractions such as lipids, polyphenols, and proteins [47,48]. No new crystalline peaks was found in any of the TBDN samples, which indicated that the addition of TBBF did not change the crystalline pattern of the starch. However, when the amount of TBBF increased, the crystal peaks at 17° and 18° (2θ) gradually became stronger (Figure 2). Table 5 shows that the crystallinity of TBDN starch significantly (*p* < 0.05) increased, from 20.15 (Control) to 24.55 (TBBF-35), an increase of 22%. The reason for this might have been that the dietary fiber competed with starch for water absorption, resulting in less contact of the starch with water and protecting the structural integrity of the starch. In addition, the dietary fiber interacted with the crystalline region of amylopectin, due to its polyhydroxy structure, which increased the density of double helix accumulation [49]. Moreover, the formation of polyphenol–starch complexes increased the crystallinity of starch [47].

### 3.5. FTIR Results of TBDNs

**Figure 3:**
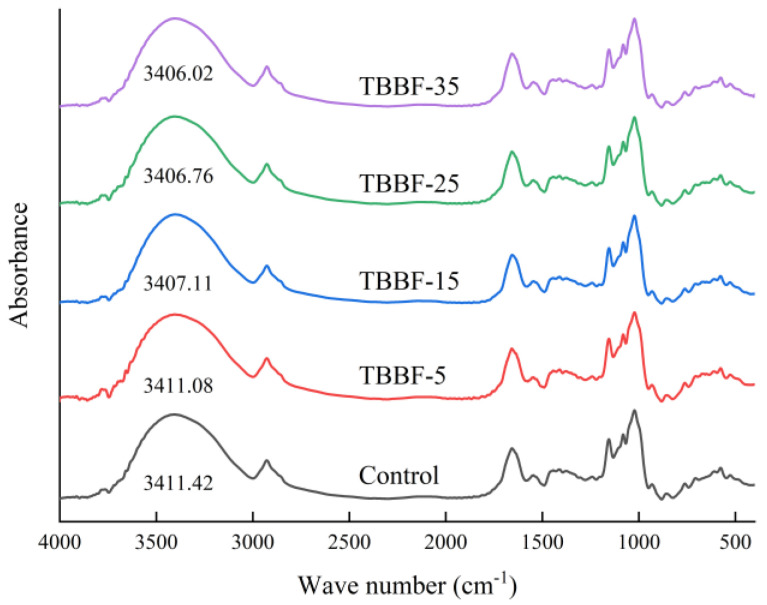
Fourier transform infrared spectra of Tartary buckwheat dried noodles. Control, TBBF-5, TBBF-15, TBBF-25, and TBBF-35 represent Tartary buckwheat dried noodles with 0%, 5%, 15%, 25%, and 35% substitution of Tartary buckwheat bran flour, respectively.

The FTIR spectra of Tartary buckwheat noodles with different TBBF substitutions are given in Figure 3. The obvious strong and broad peak at 3000–3600 cm^−1^ represents the O–H stretching vibration, the absorption peak at 2930 cm^−1^ is related to the C–H stretching vibration, and the characteristic peaks at 1200–800 cm^−1^ in the spectrum were mainly caused by the vibrations of starch polysaccharides, which could reflect the conformation of starch [50]. In particular, the absorption peak at around 1050 cm^−1^ is associated with the double helix structure of the starch crystalline region, the absorption peak at around 1022 cm^−1^ corresponds to the amorphous region of starch, and the absorption peak at around 992 cm^−1^ represents hydrogen bonding [50]. As observed in Figure 3, no new absorption peaks were formed in any of the noodle samples, suggesting that covalent interactions were not involved. However, the O–H absorption peak visibly moved to lower wave numbers, indicating the strengthening of the hydrogen bonds of the noodle systems. It was previously reported that fibers and phenolic compounds interacted with starch by the hydrogen bonds, causing the red-shift of absorption peaks [51,52].

R-1050/1022 and R-1022/992 (Table 5) were used to represent the short-range starch structure of the TBDNs. Table 5 shows that, as the substitution of TBBF rose, R-1050/1022 increased from 0.654 to 0.738, while the R-1022/992 decreased from 1.544 to 1.293. The change of R-1050/1022 and R-1022/992 confirmed that the short-range structure of TBDNs starch became more ordered, which was consistent with the crystallinity (long-range ordered) results. These results indicate an enhancement of the degree of starch structural order and hydrogen bond intensity, which likely can be ascribed to the interaction between starch and bioactive compounds in the TBBF [53].

### 3.6. Fluorescence Quenching of TBDNs

**Figure 4:**
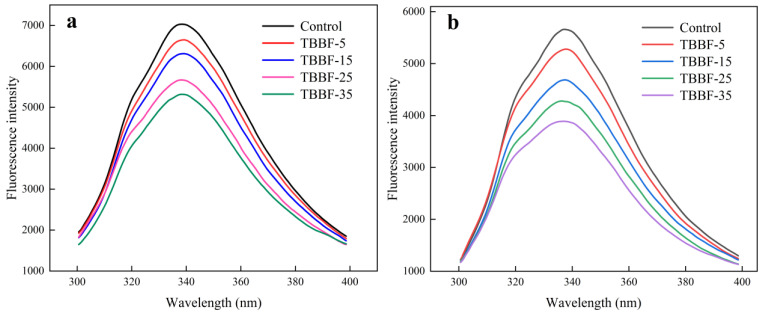
Fluorescence spectra of α-amylase (**a**) and amyloglucosidase (**b**) of Tartary buckwheat dried noodles with different substitutions of Tartary buckwheat bran flour. Control, TBBF-5, TBBF-15, TBBF-25, and TBBF-35 represent Tartary buckwheat dried noodles with 0%, 5%, 15%, 25%, and 35% substitution of Tartary buckwheat bran flour, respectively.

To further understand the mechanism by which TBBF reduced the starch digestibility, α-amylase and amyloglucosidase fluorescence quenching of different TBBF substitutions for TBDNs were studied. As shown in Figure 4, with the substitution of TBBF, the intensity of α-amylase and amyloglucosidase was gradually quenched, suggesting that the components in TBFF interacted with α-amylase and amyloglucosidase. Fluorescence quenching is due to the decrease of fluorescence quantum yield caused by molecular interactions, which respond to changes in enzyme activity [54]. The intrinsic fluorescence peak of the α-amylase and amyloglucosidase was seen at 344 nm, which is mainly related to amino acid residues of tryptophan and tyrosine [55]. Liu et al. [26] found that increasing the substitution of yam flour in bread samples decreased the intensity of α-amylase and amyloglucosidase, resulting in lower starch digestibility. Phenolic, flavonoids, and dietary fiber have been reported to quench the characteristic fluorescence of these digestive enzymes [54,55,56]. It was concluded that the substitution of TBBF increased the phenolic, flavonoids, and dietary fiber of the TBDNs (Table 3), which interacted with digestive enzymes to inhibit enzyme activity, leading to a decrease in the starch digestibility of TBDNs. Combined with the results of starch properties, the ordered structure and low digestive enzyme activity caused by the substitution of TBBF were the reason for the decrease of starch digestibility of the TBDNs.

## 4. Conclusions

The replacement of TBF with TBBF decreased the *in vitro* starch digestibility of TBDNs. Compared to the control sample, the C_∞_ and eGI values of TBBF-35 were significantly (*p* < 0.05) reduced, by 5.79% and 6.32, respectively. The LOS fitting results showed that TBBF mainly influenced the starch digestion rate of the latter part. From the evolution of the starch structure of the TBDNs, the increase in long- and short-range ordered structures resulted in more resistance to thermal processing and enzymatic digestion of TBDN starch. In addition, the fluorescence quenching intensity was enhanced as the proportion of TBBF increased. Thus, the substitution of TBBF resulted in an ordered starch structure and low enzyme activity, which reduced the starch digestibility of TBDNs. Although the addition of TBBF decreased the cooking properties, tensile strength, and elasticity of TBDNs, it increased the content of bioactive compounds and the textural attributes were still acceptable with a proper substitution of TBBF. This study proved that TBBF can be used as an ingredient to decrease the GI value of noodles.

## Figures and Tables

**Table 1:** Flour ratios of TBDNs with different substitutions of Tartary buckwheat bran flour.

	Control	TBBF-5	TBBF-15	TBBF-25	TBBF-35
Wheat flour	30	30	30	30	30
Tartary buckwheat flour	70	65	55	45	35
Tartary buckwheat bran flour	0	5	15	25	35
Total	100	100	100	100	100

TBDN: Tartary buckwheat dried noodle; Control, TBBF-5, 15, 25, and 35: Tartary buckwheat dried noodles with 0%, 5%, 15%, 25%, and 35% substitution of Tartary buckwheat bran flour.

**Table 2:** Effect of TBBF substitution on the cooking and textural properties of TBDNs.

Sample	Control	TBBF-5	TBBF-15	TBBF-25	TBBF-35
Cooking properties					
Water absorption (%)	132.80 ± 0.40 ^a^	131.81 ± 0.39 ^a^	126.65 ± 0.05 ^b^	124.16 ± 0.21 ^c^	119.02 ± 0.72 ^d^
Cooking loss (%)	4.64 ± 0.07 ^d^	4.72 ± 0.07 ^d^	5.32 ± 0.03 ^c^	5.55 ± 0.11 ^b^	6.53 ± 0.13 ^a^
Texture properties					
Hardness (g)	5547.64 ± 38.34 ^b^	5691.65 ± 35.01 ^b^	5844.12 ± 33.57 ^a^	5598.94 ± 36.44 ^b^	5415.43 ± 38.11 ^c^
Cohesiveness	0.67 ± 0.01 ^a^	0.65 ± 0.02 ^ab^	0.63 ± 0.01 ^bc^	0.62 ± 0.01 ^bc^	0.60 ± 0.02 ^c^
Resilience	0.43 ± 0.01 ^cd^	0.44 ± 0.01 ^bc^	0.48 ± 0.01 ^a^	0.46 ± 0.01 ^ab^	0.41 ± 0.02 ^d^
Tensile strength (g)	15.98 ± 0.58 ^a^	14.80 ± 0.46 ^b^	13.37 ± 0.35 ^c^	13.00 ± 0.50 ^c^	13.10 ± 0.53 ^c^
Elasticity (mm)	19.50 ± 0.73 ^a^	17.83 ± 0.79 ^a^	15.75 ± 0.94 ^b^	15.02 ± 0.76 ^bc^	12.68 ± 0.51 ^c^

Data are mean of more than three tests ± SD; Significant differences (*p* < 0.05) between parameters for the same column are represented by different lowercase letters. Control, TBBF-5, 15, 25, and 35: Tartary buckwheat dried noodles with 0%, 5%, 15%, 25%, and 35% substitution of Tartary buckwheat bran flour.

**Table 3:** Effect of TBBF substitution on the bioactive compounds content of TBDNs.

Sample			Control	TBBF-5	TBBF-15	TBBF-25	TBBF-35
TDF			3.47 ± 0.19 ^e^	4.58 ± 0.33 ^d^	6.43 ± 0.11 ^c^	8.47 ± 0.33 ^b^	9.92 ± 0.38 ^a^
IDF			2.55 ± 0.13 ^e^	3.64 ± 0.21 ^d^	5.18 ± 0.20 ^c^	6.84 ± 0.29 ^b^	8.48 ± 0.15 ^a^
SDF			0.70 ± 0.01 ^d^	0.91 ± 0.04 ^c^	1.07 ± 0.08 ^bc^	1.20 ± 0.07 ^ab^	1.37 ± 0.06 ^a^
TPC (mg GAE/g DW)		Raw noodles	5.86 ± 0.01 ^e^	6.76 ± 0.02 ^d^	8.57 ± 0.02 ^c^	10.46 ± 0.03 ^b^	12.32 ± 0.04 ^a^
		Cooked noodles	4.51 ± 0.03 ^e^	5.27 ± 0.01 ^d^	6.52 ± 0.24 ^c^	7.32 ± 0.02 ^b^	7.64 ± 0.05 ^a^
		Retention rate (%)	77	78	76	70	62
TFC (mg RE/g DW)		Raw noodles	5.36 ± 0.19 ^d^	5.52 ± 0.05 ^d^	5.93 ± 0.19 ^c^	6.77 ± 0.05 ^b^	7.95 ± 0.06 ^a^
		Cooked noodles	4.45 ± 0.28 ^c^	4.72 ± 0.05 ^c^	5.29 ± 0.15 ^b^	5.66 ± 0.05 ^b^	6.39 ± 0.37 ^a^
	Retention rate (%)		83	86	89	84	80

Data are means of more than three tests ± SD; Significant differences (*p* < 0.05) between parameters for the same column are represented by different lowercase letters; TDF, IDF, SDF: total, insoluble, soluble dietary fiber; TPC: total phenolic content; TFC: total flavonoids content; GAE: gallic acid; RE: rutin; DW: dry weight. Control, TBBF-5, 15, 25, and 35: Tartary buckwheat dried noodles with 0%, 5%, 15%, 25%, and 35% substitution of Tartary buckwheat bran flour.

**Table 4:** Effect of TBBF substitution on the digestibility of TBDNs.

Sample	k × 10^−2^ (min^−1^)	k_1_ × 10^−2^ (min^−1^)	k_2_ × 10^−2^ (min^−1^)	C_∞_ (%)	RDS (%)	SDS (%)	RS (%)	eGI
Control	1.56 ± 0.05 ^a^	3.45 ± 0.36 ^a^	1.50 ± 0.13 ^a^	54.29 ± 0.57 ^a^	16.78 ± 0.61 ^a^	27.93 ± 0.22 ^a^	55.29 ± 0.39 ^c^	59.49 ± 0.33 ^a^
TBBF-5	1.49 ± 0.06 ^ab^	3.47 ± 0.37 ^a^	1.10 ± 0.04 ^b^	52.57 ± 0.22 ^b^	16.41 ± 0.44 ^ab^	26.01 ± 0.49 ^b^	57.58 ± 0.72 ^b^	57.82 ± 0.32 ^b^
TBBF-15	1.40 ± 0.03 ^b^	3.35 ± 0.24 ^a^	0.98 ± 0.08 ^c^	50.05 ± 0.28 ^c^	14.88 ± 0.70 ^c^	23.93 ± 1.19 ^c^	61.19 ± 0.49 ^a^	54.26 ± 0.33 ^c^
TBBF-25	1.34 ± 0.04 ^b^	3.34 ± 0.30 ^a^	0.76 ± 0.02 ^d^	49.70 ± 0.42 ^c^	14.21 ± 0.49 ^c^	23.45 ± 0.50 ^c^	62.34 ± 0.50 ^a^	53.47 ± 0.22 ^d^
TBBF-35	1.35 ± 0.07 ^b^	3.81 ± 0.29 ^a^	0.61 ± 0.09 ^e^	48.50 ± 0.64 ^d^	15.29 ± 0.56 ^bc^	23.20 ± 0.56 ^c^	61.51 ± 0.51 ^a^	53.17 ± 0.22 ^d^

Data are means of more than three tests ± SD; Significant differences (*p* < 0.05) between parameters for the same column are represented by different lowercase letters; k: overall digestibility rate constant; k_1_: the higher digestibility rate constant of the first part; k_2_: the lower digestibility rate constant of the second part of digestion; C_∞_: calculated equilibrium starch hydrolysis; control, TBBF-5, 15, 25, and 35: Tartary buckwheat dried noodles with 0%, 5%, 15%, 25%, and 35% substitution of Tartary buckwheat bran flour.

**Table 5:** Effect of TBBF substitution on the thermal properties, and long- and short-range ordered structures of starch in TBDNs.

Sample	Thermal Properties				Long-Range Order	Short-Range Order	
	T_o_ (°C)	T_p_ (°C)	T_c_ (°C)	ΔH (J/g)	RC (%)	R-1050/1022	R-1022/992
Control	63.37 ± 0.29 ^d^	70.15 ± 0.10 ^a^	74.26 ± 0.28 ^a^	3.66 ± 0.20 ^a^	20.15 ± 0.65 ^d^	0.654 ± 0.001 ^d^	1.544 ± 0.003 ^a^
TBBF-5	64.36 ± 0.63 ^cd^	70.44 ± 0.12 ^a^	75.58 ± 0.04 ^a^	3.59 ± 0.02 ^a^	20.70 ± 0.40 ^cd^	0.680 ± 0.007 ^c^	1.523 ± 0.006 ^b^
TBBF-15	65.48 ± 0.17 ^bc^	70.73 ± 0.29 ^a^	75.50 ± 0.34 ^a^	3.06 ± 0.01 ^b^	22.15 ± 0.35 ^bc^	0.685 ± 0.004 ^c^	1.488 ± 0.004 ^c^
TBBF-25	65.89 ± 0.29 ^b^	70.83 ± 0.33 ^a^	75.02 ± 0.82 ^a^	2.26 ± 0.07 ^c^	22.85 ± 0.55 ^ab^	0.705 ± 0.003 ^b^	1.380 ± 0.002 ^d^
TBBF-35	67.70 ± 0.09 ^a^	70.58 ± 0.11 ^a^	75.00 ± 0.07 ^a^	1.81 ± 0.06 ^d^	24.55 ± 0.35 ^a^	0.738 ± 0.002 ^a^	1.293 ± 0.005 ^e^

Data are means of more than three tests ± SD; significant differences (*p* < 0.05) between parameters for the same column are represented by different lowercase letters; RC: relative crystallinity; R-1050/1022: the peak intensity ratios of 1050/1022 cm^−1^, R-1022/992: the peak intensity ratios of 1022/992 cm^−1^. Control, TBBF-5, 15, 25, and 35 represent Tartary buckwheat dried noodles with 0%, 5%, 15%, 25%, and 35% substitution of Tartary buckwheat bran flour.

## Data Availability

Data is contained within the article.
